# Supplementary material for: Gender, Adverse Changes in Social Engagement and Risk of Unhealthy Eating: A Prospective Cohort Study of the Canadian Longitudinal Study on Aging (2011–2021)
Source: Nutrients. 2025 Mar 13;17(6):1005. doi: 10.3390/nu17061005 (PMC11946033; doi:10.3390/nu17061005)
Supplement: Supplementary file 1 [file nutrients-17-01005-s001.zip › nutrients-3488722 Supplementary.pdf]

Gender, adverse changes in social engagement and risk of unhealthy eating:

A prospective cohort study of the Canadian Longitudinal Study on Aging (2011-2021)

Supplementary Material

Sanaz Mehranfar, MSc, Gilciane Ceolin, Ph.D., Rana Madani Civi, MSc, Heather Keller, Ph.D., RD, Rachel A. Murphy, Ph.D., Tamara R. Cohen, Ph.D., RD, Annalijn I. Conklin, Ph.D. (Cantab), M.P.H

Table of contents:

Supplemental Figure S1. Flow diagram of the process of sample selection from the Canadian Longitudinal Study on Aging (2011-21).

Supplemental Figure S2. Directed Acyclic Graph (DAG) for social isolation (a) and social participation (diversity of social participation) (b) and fruit and vegetable intake.

Supplemental Figure S3. Average predicted probability of non-daily vegetable intake associated with social isolation transitions and social participation transitions among women and men in the Canadian Longitudinal Study on Aging (2011-21).

Supplemental Figure S4. Average predicted probability of non-daily fruit intake associated with social isolation transitions and social participation transitions among women and men in the Canadian Longitudinal Study on Aging (2011-21).

Supplemental Figure S5. Frequency of social activities across social isolation transitions using analytic sample size of vegetable among women.

Supplemental Figure S6. Frequency of social activities across social isolation transitions using analytic sample size of fruit among women.

Supplemental Figure S7. Frequency of social activities across social participation transitions using analytic sample size of fruit among women.

Supplemental Table S1. Coding process of mock examples of dietary data from the CLSA Short Diet Questionnaire.

Supplemental Table S2. Sample characteristics across social isolation transitions and social participation transitions among aging women and men in the eligible sample consuming daily fruit intake in the Canadian Longitudinal Study on Aging (2011-21).

Supplemental Table S3. Sensitivity analysis of the associations between social isolation transitions and non-daily vegetable intake among aging women and men in the Canadian Longitudinal Study on Aging (2011-21).

Supplemental Table S4. Sensitivity analysis of the associations between social participation transitions and non-daily vegetable intake among aging women and men in the Canadian Longitudinal Study on Aging (2011-21).

Supplemental Table S5. Sensitivity analysis of the associations between social isolation transitions and non-daily fruit intake among aging women and men in the Canadian Longitudinal Study on Aging (2011-21).

Supplemental Table S6. Sensitivity analysis of the associations between social participation transitions and non-daily fruit intake among aging women and men in the Canadian Longitudinal Study on Aging (2011-21).

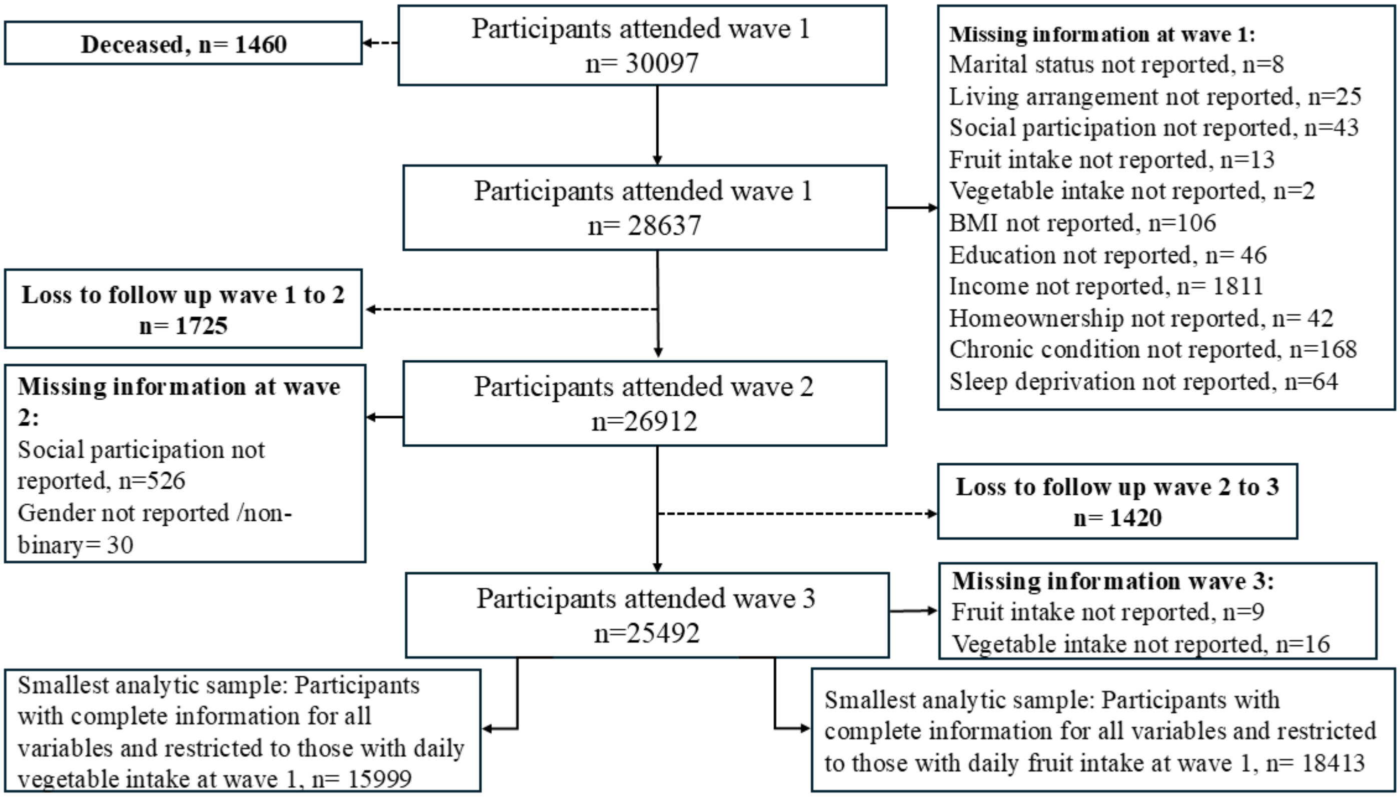

Supplemental Figure S1. Flow diagram of the process of sample selection from the Canadian Longitudinal Study on Aging (2011-21).

Supplemental Table S1. Coding process of mock examples of dietary data from the CLSA Short Diet Questionnaire.

Entity ID	Items	Per year/ Never	Per month	Per week	Per day	CLSA data (times per day)	Daily or non-daily
**#1**	Fruit			7		1	Daily
**#1**	Green salad				2	2	Daily
**#1**	Potatoes		15			0.5	Non-daily
**#1**	Carrots			5		0.71	Non-daily
**#1**	Other vegetables			14		2	Daily
**#2**	Fruit				5	5	Daily
**#2**	Green salad		25			0.83	Non-daily
**#2**	Potatoes			2		0.29	Non-daily
**#2**	Carrots	0				0	Non-daily
**#2**	Other vegetables			20		2.86	Daily

CLSA question was "How often do you usually eat each food group? For example, twice a day, three times a week, once a month?" CLSA converted frequency responses to times per day using 7 as the denominator for weekly intake and 30 for monthly.

A:

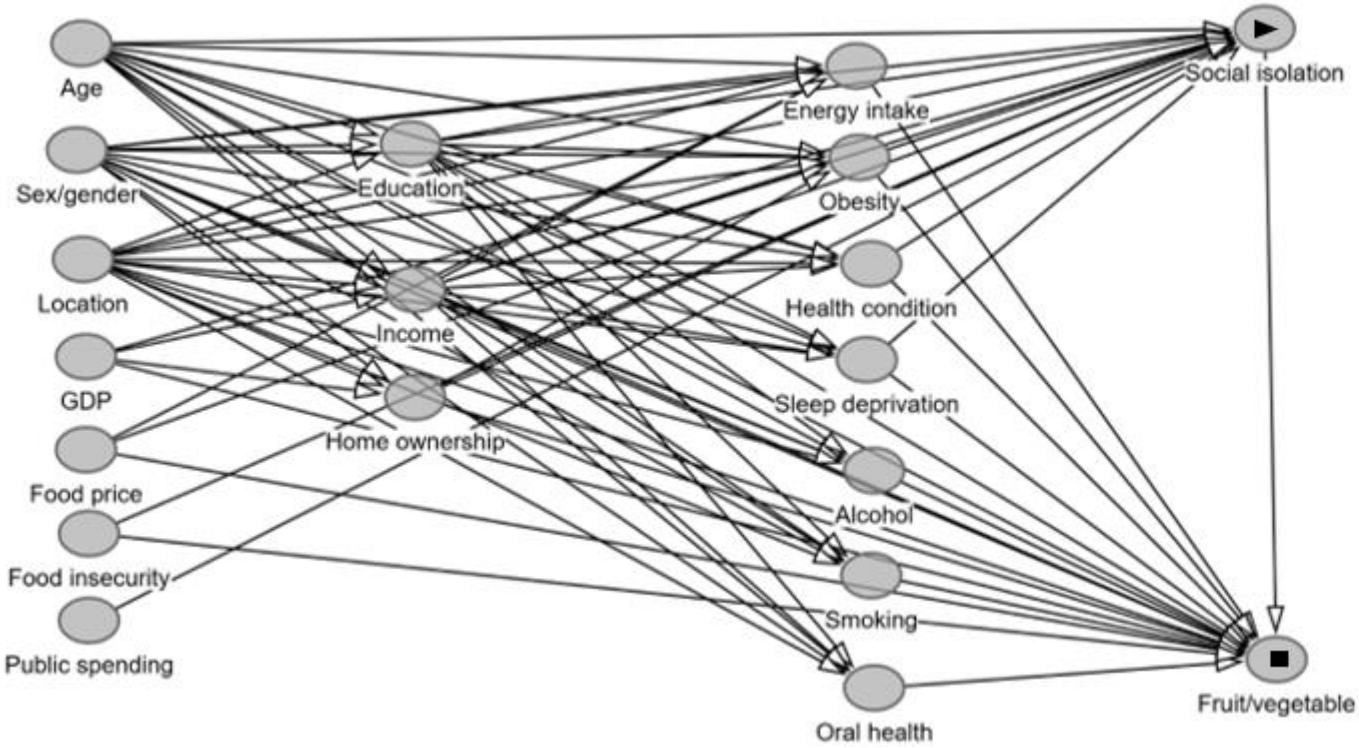

B:

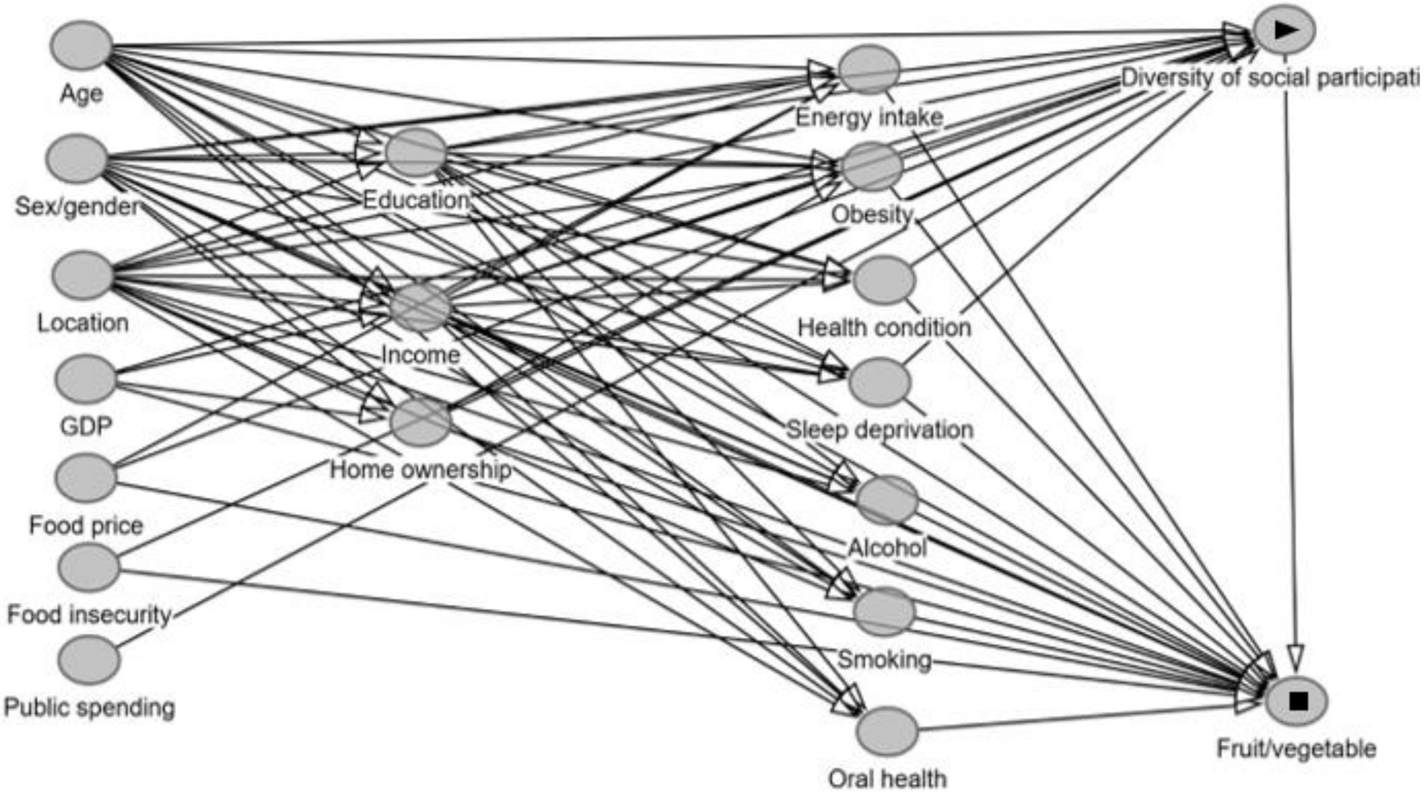

Supplemental Figure S2. Directed Acyclic Graph (DAG) for social isolation (a) and social participation (diversity of social participation) (b) and fruit and vegetable intake. The minimum set of adjustments covariates for confounding was constructed based on the associations described in the literature to test the association between social isolation /social participation and FV intake. The exposures are social isolation or social participation, and the outcome variable is FV intake. Each variable is represented by the circles; the arrows represent the causal association between each variable. GDP, Gross domestic Product. The exposures are social isolation and diversity of social participation (play symbol) and the outcome variable is the fruit/vegetable (stop symbol).

Supplemental Table S2. Sample characteristics across social isolation transitions and social participation transitions among aging women and men in the eligible sample consuming daily fruit intake in the Canadian Longitudinal Study on Aging (2011-21).

	Age (years)	Highest education level[1]	Highest income level[2]	Home-owner	Urban location	BMI (kg/m²)	No chronic condition	Not sleep deprived	Non-daily fruit intake
**Women**									
Remained not isolated (n=9586)	59.23 (9.86)	7678 (67.8%)	1486 (17.1%)	8304 (87.4%)	8658 (92.5%)	27.6 (5.83)	511 (6.7%)	6097 (63.3%)	1269 (15.3%)
Remained isolated (n=173)	62.55 (10.71)	109 (46.8%)	9 (4.8%)	116 (67.7%)	154 (94.9%)	27.29 (6.06)	6 (3.5%)	100 (48.1%)	49 (29.0%)
Became isolated (n=384)	60.3 (10.91)	272 (48.8%)	39 (8.7%)	300 (80.8%)	348 (95.5%)	29.58 (7.11)	10 (3.9%)	198 (52.5%)	62 (14.4%)
Became not isolated (n=273)	58.55 (8.77)	174 (44.9%)	26 (9.0%)	209 (69.0%)	246 (94.9%)	28.32 (6.53)	12 (13.8%)	154 (53.1%)	64 (22.5%)
Remained diverse (n=3485)	60.84 (10.3)	2934 (73.3%)	561 (18.4%)	3088 (89.1%)	3169 (92.8%)	27.36 (5.71)	174 (6.7%)	2276 (66.0%)	358 (10.4%)
Remained less diverse (n=4212)	58.44 (9.65)	3104 (60.9%)	589 (14.4%)	3507 (83.3%)	3762 (92.7%)	28.01 (6.19)	213 (7.3%)	2549 (57.5%)	720 (18.8%)
Became less diverse (n=1470)	59.05 (9.99)	1183 (65.7%)	222 (16.8%)	1251 (87.7%)	1340 (92.1%)	27.75 (5.84)	83 (5.0%)	920 (66.2%)	207 (17.6%)
Became diverse (n=1249)	59.87 (9.52)	1012 (66.6%)	188 (16.9%)	1083 (87.8%)	1135 (93.9%)	27.31 (5.53)	69 (6.4%)	804 (66.2%)	159 (14.0%)
**Men**									
Remained not isolated (n=7164)	58.87 (9.9)	6,032 (70.7%)	1630 (23.2%)	6492 (87.7%)	6544 (94.4%)	28.04 (4.57)	574 (10.3%)	4641 (63.3%)	1440 (20.8%)
Remained isolated (n=201)	61.19 (8.89)	145 (64.1%)	14 (4.6%)	159 (76.4%)	183 (95.5%)	28.35 (5.72)	11 (8.8%)	110 (57.5%)	54 (24.9%)
Became isolated (n=363)	57.65 (9.54)	290 (53.4%)	48 (14.3%)	295 (84.1%)	318 (94.7%)	29.09 (5.92)	30 (6.4%)	224 (66.5%)	92 (36.9%)
Became not isolated (n=269)	59.04 (10.18)	201 (55.2%)	41 (12.1%)	223 (75.3%)	244 (94.7%)	29.39 (5.1)	26 (7.1%)	168 (60.5%)	65 (26.4%)
Remained diverse (n=2305)	60.72 (10.49)	2025 (74.8%)	535 (23.9%)	2103 (91.3%)	2110 (93.6%)	28.47 (4.57)	181 (10.8%)	1529 (63.5%)	416 (16.8%)
Remained less diverse (n=3778)	57.96 (9.45)	3018 (64.9%)	753 (19.9%)	3317 (83.2%)	3415 (94.4%)	27.94 (4.81)	313 (10.0%)	2378 (62.6%)	864 (25.4%)
Became less diverse (n=1044)	58.9 (10)	895 (71.6%)	257 (25.3%)	960 (90.9%)	961 (95.1%)	28.37 (4.68)	78 (9.0%)	661 (65.6%)	203 (19.1%)
Became diverse (n=870)	59.33 (9.87)	730 (72.6%)	188 (21.7%)	789 (88.9%)	803 (96.0%)	28.43 (4.62)	69 (8.6%)	575 (62.9%)	168 (20.9%)

Descriptive statistics, including percentages (%) and means (SD), were calculated using CLSA survey inflation weights. All variables were reported at baseline, except for fruit intake at follow-up 2. 1 The highest education level was post-secondary graduation [university degree]. 2 The highest income was ≥ CAD 150,000.

Supplemental Table S3. Sensitivity analysis of the associations between social isolation transitions and non-daily vegetable intake among aging women and men in the Canadian Longitudinal Study on Aging (2011-21).

	Model A: exclude potato[a]		Model B: + oral health		Model C: + smoking		Model D: + alcohol consumption		Model E: + reproductive factors[b]		Model F: + social ties[c]		Model G: + exclude those interviewed during Covid-19	
	**OR**	**CI95**	**OR**	**CI95**	**OR**	**CI95**	**OR**	**CI95**	**OR**	**CI95**	**OR**	**CI95**	**OR**	**CI95**
Women														
Remained isolated	1.69**	[1.19, 2.40]	1.88***	[1.34, 2.65]	1.81**	[1.29, 2.54]	1.82**	[1.29, 2.57]	1.77**	[1.25, 2.5]	1.71**	[1.22, 2.41]	1.63*	[1.04, 2.55]
Became isolated	1.18	[0.91, 1.52]	1.15	[0.89, 1.49]	1.18	[0.92, 1.53]	1.19	[0.92, 1.54]	1.16	[0.9, 1.51]	1.14	[0.88, 1.47]	1.34	[0.98, 1.84]
Became not isolated	1.30	[0.97, 1.75]	1.31	[0.97, 1.76]	1.36*	[1.02, 1.82]	1.38*	[1.02, 1.85]	1.35*	[1.01, 1.81]	1.30	[0.97, 1.74]	1.51*	[1.05, 2.17]
Men														
Remained isolated	1.09	[0.79, 1.51]	1.13	[0.83, 1.55]	1.13	[0.82, 1.55]	1.11	[0.81, 1.53]	-	-	1.06	[0.77, 1.45]	1.24	[0.84, 1.84]
Became isolated	1.11	[0.86, 1.44]	1.11	[0.86, 1.43]	1.15	[0.89, 1.48]	1.18	[0.91, 1.51]	-	-	1.11	[0.86, 1.43]	1.54**	[1.12, 2.13]
Became not isolated	1.30	[0.97, 1.73]	1.27	[0.96, 1.69]	1.31	[0.98, 1.74]	1.33	[1.00, 1.76]	-	-	1.26	[0.94, 1.67]	1.72**	[1.21, 2.43]

Gender-specific odds ratios (95% CIs) obtained by mixed-effects logistic regression with an interaction term (gender x changes in social isolation) on the sample with baseline daily intake. Reference is, remained not isolated. Model A excludes potato from total vegetable intake (n=15767). Model B further adjusts for oral health (n=15795). Model C adjusts for smoking (n=15999). Model D adjusts for alcohol consumption (n=15649). Model E includes parity, menopause, and hormone replacement therapy (n=9288). Model F also includes baseline social network, marital status, living arrangement (n=15995). Model G excludes those who were interviewed during COVID-19 lockdown (n=10310). *p<0.05; ** p<0.01; *** p<0.001.

Supplemental Table S4. Sensitivity analysis of the associations between social participation transitions and non-daily vegetable intake among aging women and men in the Canadian Longitudinal Study on Aging (2011-21).

	Model A: exclude potato[a]		Model B: + oral health		Model C: + smoking		Model D: + alcohol consumption		Model E: + reproductive factors[b]		Model F: + social ties[c]		Model G: + exclude those interviewed during Covid-19	
	**OR**	**CI95**	**OR**	**CI95**	**OR**	**CI95**	**OR**	**CI95**	**OR**	**CI95**	**OR**	**CI95**	**OR**	**CI95**
Women														
Remained less diverse	1.31***	[1.15, 1.49]	1.27***	[1.12, 1.45]	1.28***	[1.13, 1.46]	1.29***	[1.13, 1.46]	1.32***	[1.16, 1.51]	1.24**	[1.09, 1.41]	1.25**	[1.07, 1.47]
Became less diverse	1.12	[0.94, 1.33]	1.07	[0.9, 1.27]	1.08	[0.91, 1.28]	1.08	[0.91, 1.29]	1.10	[0.93, 1.32]	1.07	[0.89, 1.27]	1.16	[0.94, 1.44]
Became diverse	1.11	[0.92, 1.33]	1.08	[0.90, 1.30]	1.09	[0.91, 1.31]	1.10	[0.91, 1.32]	1.10	[0.91, 1.33]	1.07	[0.88, 1.28]	0.99	[0.79, 1.24]
Men														
Remained less diverse	1.26***	[1.11, 1.43]	1.28***	[1.12, 1.46]	1.28***	[1.12, 1.46]	1.28***	[1.13, 1.46]	-	-	1.21**	[1.06, 1.38]	1.25**	[1.06, 1.46]
Became less diverse	1.04	[0.87, 1.25]	1.03	[0.85, 1.24]	1.03	[0.85, 1.23]	1.04	[0.86, 1.25]	-	-	0.99	[0.82, 1.19]	0.94	[0.74, 1.19]
Became diverse	1.19	[0.98, 1.44]	1.15	[0.95, 1.40]	1.15	[0.95, 1.39]	1.16	[0.95, 1.41]	-	-	1.12	[0.92, 1.35]	1.06	[0.83, 1.34]

Gender-specific odds ratios (95% CIs) obtained by mixed-effects logistic regression with an interaction term (gender x changes in diversity of social participation) on the sample with baseline daily intake. Reference is, remained diverse. Model A excludes potato from total vegetable intake (n=15767). Model B further adjusts for oral health (n=15795). Model C adjusts for smoking (n=15999). Model D adjusts for alcohol consumption (n=15649). Model E includes parity, menopause, and hormone replacement therapy (n=9288). Model F also includes baseline social network, marital status, living arrangement (n=15995). Model G excludes those who were interviewed during COVID-19 lockdown (n=10310). $^{**}p<0.01$; $^{***}p<0.001$.

A

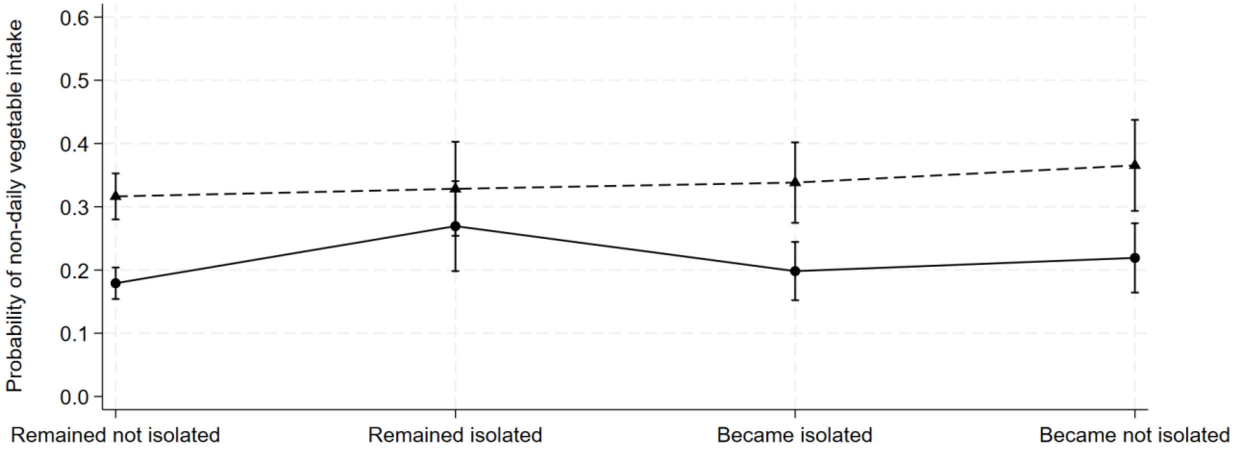

B

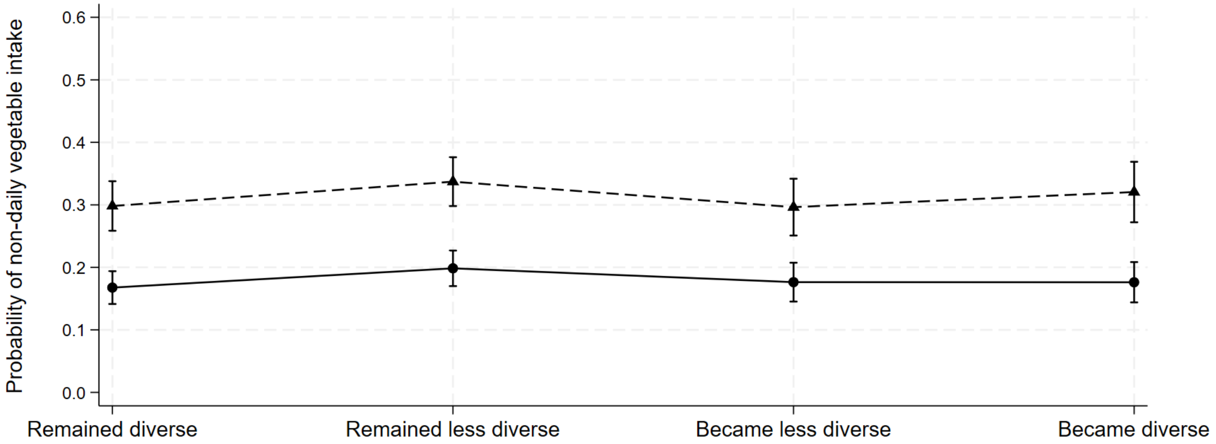

Supplemental Figure S3. Average predicted probability of non-daily vegetable intake associated with social isolation transitions and social participation transitions among women and men in the Canadian Longitudinal Study on Aging (2011-21), independent of other social ties (full model (adjusted for follow-up time, age, BMI, chronic condition, education, income, home-ownership, geographic location, sleep deprivation, provincial variables) + adjustment for baseline social network, marital status, living arrangement). Dash line, men; solid line, women. Panel A, social isolation transitions; Panel B, social participation transitions. The social isolation transition variable is classified as: (1) remained isolated (no change in 0 or 1 activity/month at both waves); (2) remained not isolated (no change in 2 or more activities/month at both waves); (3) became isolated (transition to 0 or 1 activity/month); and (4) became not isolated (transition to 2 or more activities/month). Social participation transitions are classified as: (1) remained diverse (no change in ≥5 activities/month at both waves); (2) remained less diverse (no change in <5 activities/month at both waves); (3) became less diverse (transition from ≥5 activities to <5 activities/month); and (4) became diverse (transition from <5 activities to ≥5 activities/month).

Supplemental Table S5. Sensitivity analysis of the associations between social isolation transitions and non-daily fruit intake among aging women and men in the Canadian Longitudinal Study on Aging (2011-21).

	Model A: + oral health		Model B: + smoking		Model C: + alcohol consumption		Model D: + reproductive factors[b]		Model E: + social ties[c]		Model F: + exclude those interviewed during Covid-19	
	**OR**	**CI95**	**OR**	**CI95**	**OR**	**CI95**	**OR**	**CI95**	**OR**	**CI95**	**OR**	**CI95**
Women												
Remained isolated	2.27***	[1.60, 3.20]	2.13***	[1.51, 3.01]	2.35***	[1.66, 3.33]	2.34***	[1.66, 3.32]	2.07***	[1.47, 2.92]	2.80***	[1.83, 4.28]
Became isolated	1.12	[0.84, 1.48]	1.11	[0.83, 1.47]	1.13	[0.85, 1.51]	1.14	[0.86, 1.53]	1.08	[0.81, 1.43]	1.24	[0.87, 1.77]
Became not isolated	1.68**	[1.25, 2.26]	1.74**	[1.30, 2.33]	1.83***	[1.36, 2.46]	1.77***	[1.32, 2.38]	1.68***	[1.25, 2.25]	1.98***	[1.38, 2.82]
Men												
Remained isolated	1.38	[1.00, 1.91]	1.33	[0.97, 1.84]	1.36	[0.98, 1.89]	-	-	1.25	[0.90, 1.73]	1.19	[0.78, 1.82]
Became isolated	1.27	[0.99, 1.63]	1.24	[0.96, 1.58]	1.32	[1.03, 1.69]	-	-	1.22	[0.95, 1.56]	1.35	[0.98, 1.86]
Became not isolated	1.16	[0.86, 1.55]	1.16	[0.87, 1.55]	1.19	[0.89, 1.59]	-	-	1.11	[0.83, 1.48]	1.02	[0.71, 1.48]

Gender-specific odds ratios (95% CIs) obtained by mixed-effects logistic regression with an interaction term (gender x changes in social isolation) on the sample with baseline daily intake. Reference is, remained not isolated. Model A further adjusts for oral health (n=18192). Model B adjusts for smoking (n=18413). Model C adjusts for alcohol consumption (n=17992). Model D includes parity, menopause, hormone replacement therapy (n=10287). Model E also includes BL social network, marital status, and living arrangement (n=18407). Model F excludes those who were interviewed during COVID-19 lockdown (n=11945). *p<0.05; ** p<0.01; *** p<0.001.

Supplemental Table S6. Sensitivity analysis of the associations between social participation transitions and non-daily fruit intake among aging women and men in the Canadian Longitudinal Study on Aging (2011-21).

	Model A: + oral health		Model B: + smoking		Model C: + alcohol consumption		Model D: + reproductive factors[b]		Model E: + social ties[c]		Model F: + exclude those interviewed during Covid-19	
	**OR**	**CI95**	**OR**	**CI95**	**OR**	**CI95**	**OR**	**CI95**	**OR**	**CI95**	**OR**	**CI95**
Women												
Remained less diverse	1.61***	[1.40, 1.86]	1.60***	[1.39, 1.83]	1.63***	[1.42, 1.88]	1.62***	[1.41, 1.86]	1.56***	[1.36, 1.80]	1.58***	[1.34, 1.88]
Became less diverse	1.33**	[1.11, 1.60]	1.33**	[1.10, 1.60]	1.30**	[1.08, 1.57]	1.34**	[1.11, 1.61]	1.33**	[1.10, 1.60]	1.28**	[1.01, 1.61]
Became diverse	1.21	[0.99, 1.48]	1.20	[0.98, 1.47]	1.20	[0.98, 1.47]	1.17	[0.96, 1.44]	1.18	[0.97, 1.45]	1.22	[0.96, 1.56]
Men												
Remained less diverse	1.28***	[1.12, 1.46]	1.27***	[1.11, 1.45]	1.30***	[1.14, 1.49]	-	-	1.21**	[1.06, 1.39]	1.25**	[1.06, 1.47]
Became less diverse	1.06	[0.88, 1.28]	1.06	[0.88, 1.28]	1.06	[0.88, 1.29]	-	-	1.04	[0.86, 1.26]	0.92	[0.72, 1.17]
Became diverse	1.07	[0.88, 1.31]	1.06	[0.87, 1.30]	1.08	[0.88, 1.32]	-	-	1.03	[0.84, 1.26]	1.01	[0.79, 1.30]

Gender-specific odds ratios (95% CIs) obtained by mixed-effects logistic regression with an interaction term (gender x changes in diversity of social participation) on the sample with baseline daily intake. Reference is, remained diverse. Model A further adjusts for oral health (n=18192). Model B adjusts for smoking (n=18413). Model C adjusts for alcohol consumption (n=17992). Model D includes parity, menopause, hormone replacement therapy (n=10287). Model E also includes BL social network, marital status, and living arrangement (n=18407). Model F excludes those who were interviewed during COVID-19 lockdown (n=11945). *p<0.05; ** p<0.01; *** p<0.001.

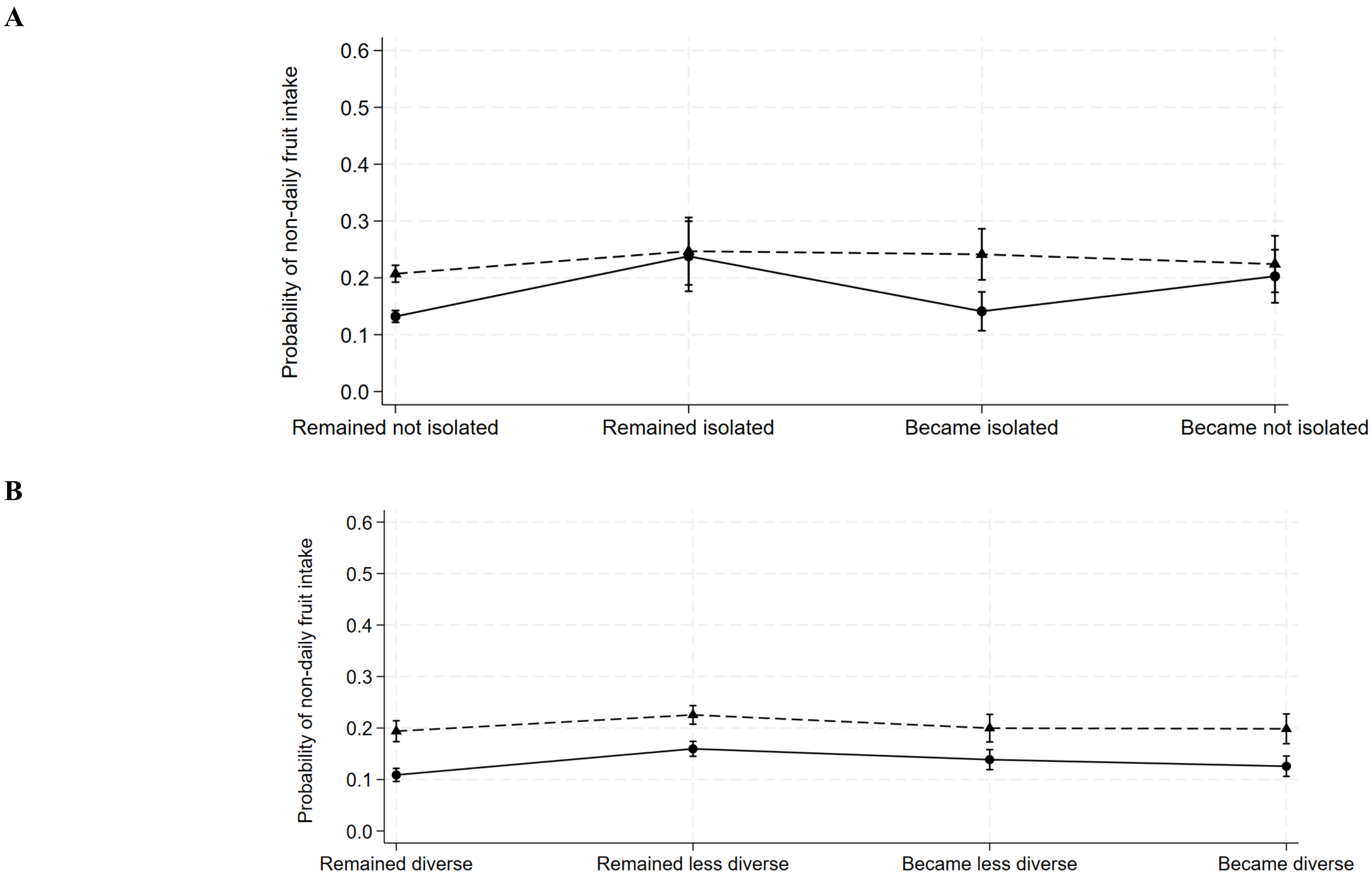

Supplemental Figure S4. Average predicted probability of non-daily fruit intake associated with social isolation transitions and social participation transitions among women and men in the Canadian Longitudinal Study on Aging (2011-21), independent of other social ties (full model (adjusted for follow-up time, age, BMI, chronic condition, education, income, home-ownership, geographic location, sleep deprivation, provincial variables) + adjustment for baseline social network, marital status, living arrangement). Dash line, men; solid line, women. Panel A, social isolation transitions; Panel B, social participation transitions. The social isolation transition variable is classified as: (1) remained isolated (no change in 0 or 1 activity/month at both waves); (2) remained not isolated (no change in 2 or more activities/month at both waves); (3) became isolated (transition to 0 or 1 activity/month); and (4) became not isolated (transition to 2 or more activities/month). Social participation transitions are classified as: (1) remained diverse (no change in ≥5 activities/month at both waves); (2) remained less diverse (no change in <5 activities/month at both waves); (3) became less diverse (transition from ≥5 activities to <5 activities/month); and (4) became diverse (transition from <5 activities to ≥5 activities/month).

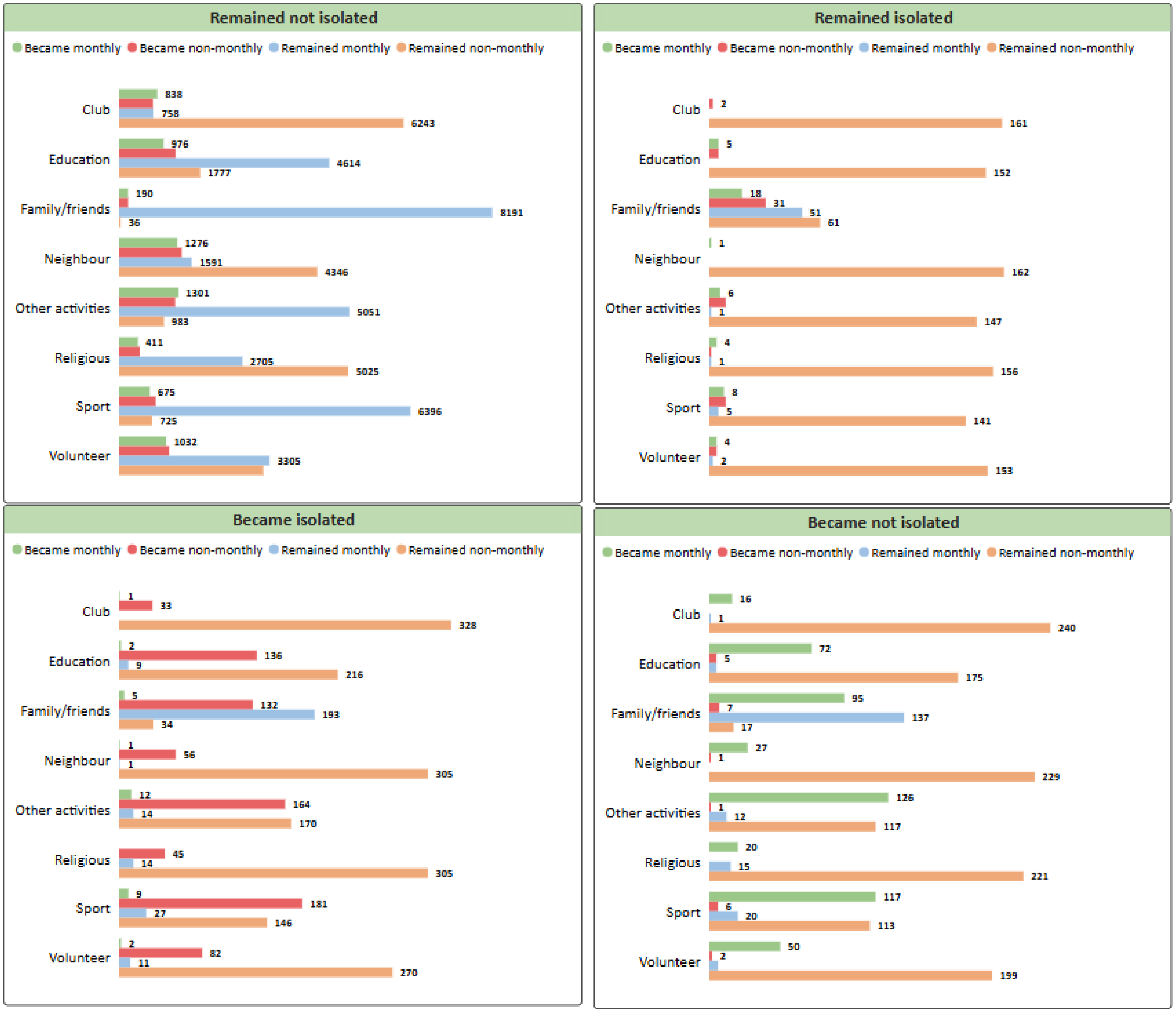

Supplemental Figure S5. Frequency of social activities across social isolation transitions using analytic sample size of vegetable among women.

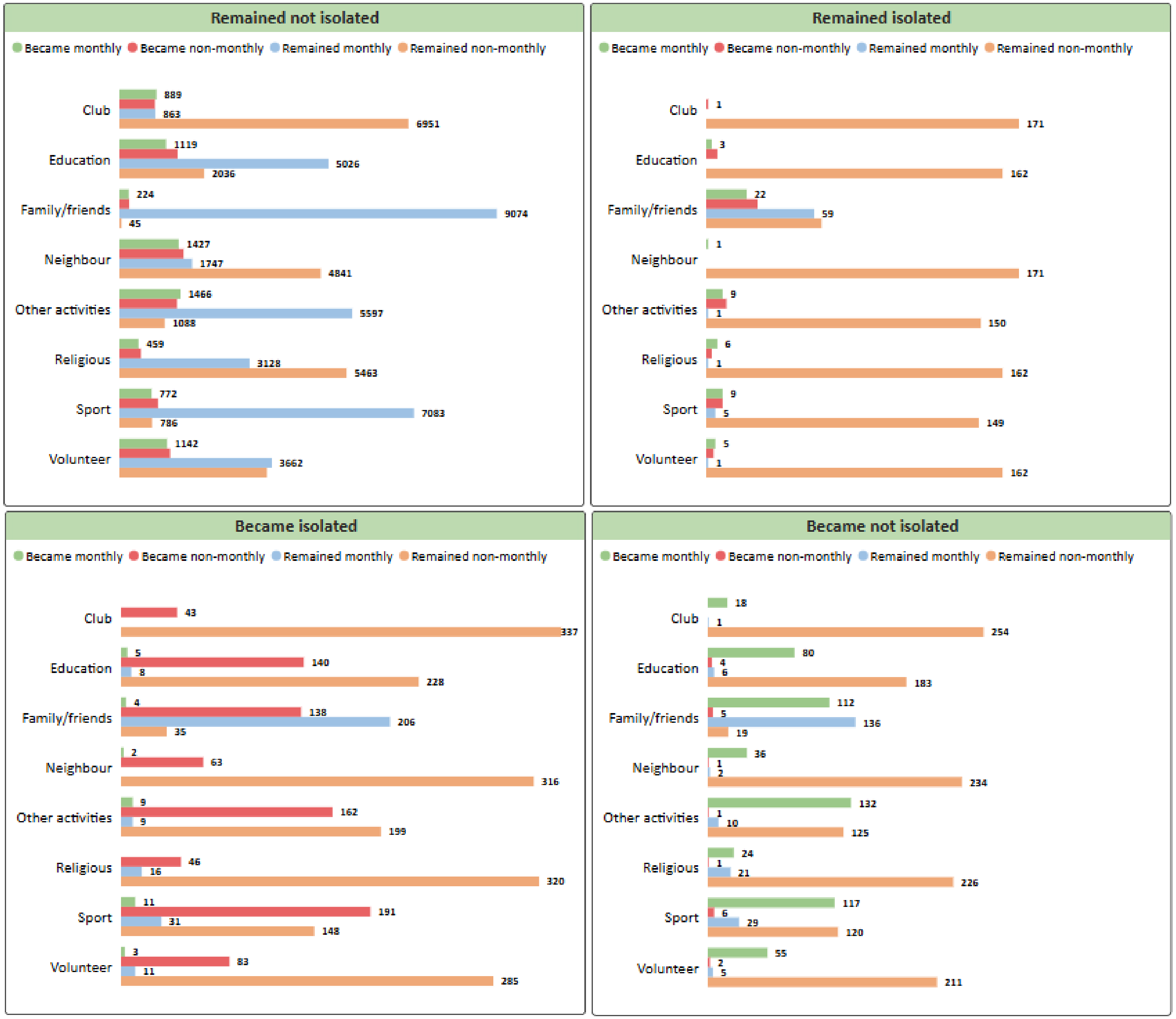

Supplemental Figure S6. Frequency of social activities across social isolation transitions using analytic sample size of fruit among women.

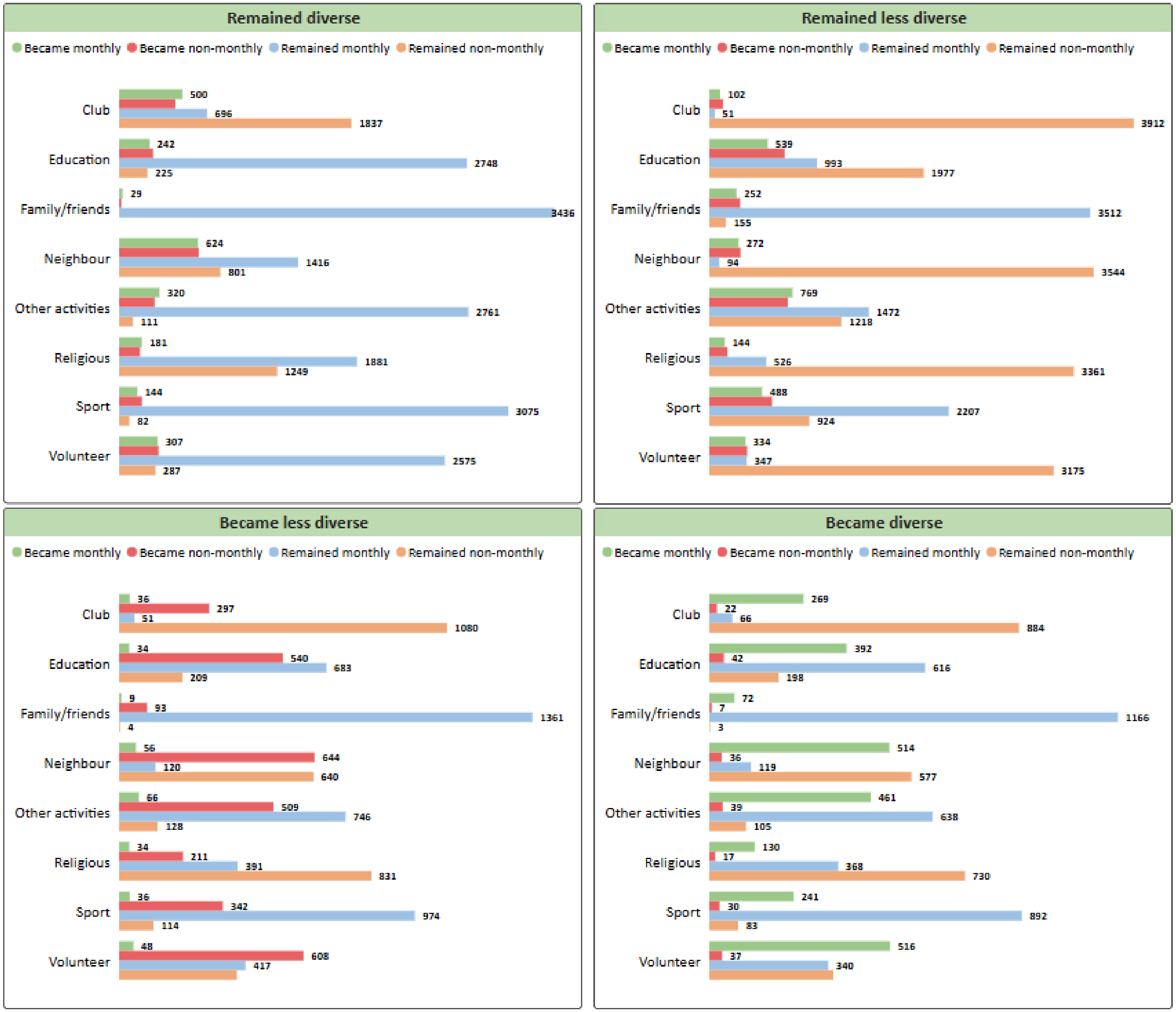

Supplemental Figure S7. Frequency of social activities across social participation transitions using analytic sample size of fruit among women.
